# Current Concepts in Pediatric Orthopedics

**DOI:** 10.2174/1874325001711010335

**Published:** 2017-04-28

**Authors:** Siddhartha Sharma, Nirmal Raj Gopinathan

**Affiliations:** Postgraduate Institute of Medical Education and Research, Department of Orthopedic Surgery, Chandigarh, India 160012

The term orthopedics has been derived from the French word ‘orthopedie’, which was coined by the French physician Nicolas Andry de Bois-Regard and literally means ‘straight child’. In 1742, Andry published his book titled *‘L’orthopédie ou l’art de prévenir et corriger dans les enfants les difformités du corp’*, which was later translated into English as ‘Orthopaedia or the art of correcting and preventing deformities in children’ and became an immediate success [1]. Over time, orthopedics has seen giant leaps in all areas, and many subspecialties have emerged, including traumatology, spinal disorders, arthroplasty, arthroscopy, hand surgery as well as foot and ankle disorders. However, management of orthopedic disorders in the pediatric age group continues to pose a significant challenge. It has been often quoted that children are not small adults, and although children can be afflicted by the same spectrum of disorders as adults, the principles of management and outcomes are very different from adults. Perhaps the biggest challenge in pediatric orthopedics is to obtain a good long-term outcome.

 What may seem like a rational treatment for a child in short-term may often prove to be ineffective or even counter-productive at long-term follow-up. Fortunately, pediatric orthopedics rests on the shoulders of giants like Mihran O. Tachdjian, Robert Salter, Walter Blount, Lynn T Staheli, John A. Herring and many others. As our understanding of pediatric orthopedics continues to evolve, it is imperative that orthopedic surgeons should develop an in-depth understanding of problems unique to the pediatric age group so as to provide the best treatment options. For some problems, all that may be necessary is observation and reassurance, as may be case for intoeing [2] and flexible flatfoot [3].

This special issue of the open orthopedics journal is devoted to some of the important musculoskeletal conditions in children and covers pediatric fractures, Perthes disease, pediatric anterior cruciate ligament injuries and congenital scoliosis. The editors hope that the articles would benefit orthopedic trainees, general orthopedic surgeons and pediatric orthopedic surgeons alike.
